# A structural equation model to assess the pathways of body adiposity and inflammation status on dysmetabolic biomarkers via red cell distribution width and mean corpuscular volume: a cross-sectional study in overweight and obese subjects

**DOI:** 10.1186/s12944-020-01308-5

**Published:** 2020-06-26

**Authors:** Mariangela Rondanelli, Simone Perna, Tariq A. Alalwan, Roberta Cazzola, Clara Gasparri, Vittoria Infantino, Federica Perdoni, Giancarlo Iannello, Daniele Pepe, Davide Guido

**Affiliations:** 1 IRCCS Mondino Foundation, 27100 Pavia, Italy; 2 Department of Public Health, Experimental and Forensic Medicine, University of Pavia, 27100 Pavia, Italy; 3 Department of Biology, College of Science, University of Bahrain, Sakhir Campus, P. O. Box 32038, Zallaq, Kingdom of Bahrain; 4 Department of Biomedical and Clinical Sciences “L. Sacco”, University of Milan, Milan, Italy; 5 Endocrinology and Nutrition Unit, Azienda di Servizi alla Persona “Istituto Santa Margherita”, University of Pavia, 27100 Pavia, Italy; 6 Department of Biomedical Science and Human Oncology, University of Bari, 70121 Bari, Italy; 7 General Management, Azienda di Servizi alla Persona “Istituto Santa Margherita”, 27100 Pavia, Italy; 8 Hasselt University, I-BioStat, Diepenbeek, Belgium; 9 Neurology, Public Health, Disability Unit, Scientific Department, Fondazione IRCCS Istituto Neurologico Carlo Besta, Milan, Italy; 10 Epidemiology Unit, Agency for Health Protection of Milan, Milan, Italy

**Keywords:** Obesity, Inflammation, Metabolism, Pathway, Red cell distribution width, Mean corpuscular volume, Reactive oxygen species, Path analysis, Structural equation modelling

## Abstract

**Background:**

A study has been performed in overweight and obese subjects to assess the effects of adiposity and inflammation indicators on dysmetabolic biomarkers via red cell distribution width (RDW) and mean corpuscular volume (MCV), taking into account pro-antioxidant balance.

**Methods:**

Data from 166 overweight subjects were analyzed by a path analysis model using structural equation modelling (SEM) to evaluate the direct and indirect pathway effects of adiposity, measured by body mass index (BMI) and waist circumference (WC), and inflammation status, measured by pro-antioxidant balance [reactive oxygen species (ROS)], lag-time and slope and C-reactive protein (CRP) values on dysmetabolic biomarkers, via RDW and MCV.

**Results:**

BMI was strongly linked to CRP and ROS levels. Moreover, there was a significant negative decrease of MCV (1.546 femtoliters) linked to BMI indirectly via high CRP levels. Furthermore, WC affected RDW, indicating a possible mediatory role for RDW in relation to the relationship between WC and homeostatic model assessment (HOMA), insulin and high density lipoprotein (HDL), respectively. This was evident by the elevated HOMA and insulin levels and the decreased levels of HDL. Finally, ROS-related markers did not affect directly RDW and MCV.

**Conclusion:**

The reported outcomes suggest that RDW might play a mediatory role in the relationship between WC and the dysmetabolic outcomes in overweight and obese individuals. CRP seems to modulate the linkage between BMI and MCV. This study provides the backbone structure for future scenarios and lays the foundation for further research on the role of RDW and MCV as suitable biomarkers for the assessment of cardiovascular disease (HDL-cholesterol), inflammatory bowels and insulin resistance.

## Introduction

Red blood cell distribution width (RDW), a measure of the variability in size of circulating erythrocytes, has been demonstrated to be altered under various disease conditions linked to inflammation [1], including chronic heart failure (CHF), pulmonary embolism, septic shock [2, 3] and haematological disease, in particular certain forms of anaemia [4]. Evidence suggest that in addition to inflammation, elevated levels of RDW are also linked to nutritional habits [5], both representing the two main characteristics of insulin resistance in obesity, and metabolic syndrome (MetS) as previously reported in the ibermutuamur cardiovascular risk assessment (ICARIA) study [6].

It is now well recognized that chronic low-grade inflammatory state is a crucial factor that links obesity to insulin resistance and its comorbidities [7]. Consequently, recent studies have focused on investigating the relationship between RDW and inflammation in obese subjects with conflicting results. For instance, RDW differed significantly between normal-weight adolescents and overweight adolescents and RDW was positively correlated with biomarkers of inflammation [8]. Moreover, in an animal model of diet-induced obesity, nutritional changes increased RDW, while overweight by definition did not alter RDW [8]. In contrast, the study by Vaya et al. pointed out that RDW should not be considered as an inflammatory marker in morbidly obese patients as it is not related to inflammatory status, in spite of reporting elevated RDW in morbidly obese patients compared to controls [9]. Inflammation and oxidative damage are correlated in obesity and it has been theorized that not only inflammation, but also oxidative damage may affect RDW, as previously demonstrated in older patients [10]. Moreover, oxidative stress increases the level of RDW by impairing iron metabolism, reducing red cell life span, and modulating the response to erythropoietin by the bone marrow [11]. Consequently, oxidative stress may be a potential underlying biological mechanism for increased RDW, however, there are no studies to date evaluating the correlation between oxidative stress and RDW in obese patients.

Given this background, the aim of the study was to assess the links between RDW and the markers of adiposity [assessed by body mass index (BMI) and waist circumference (WC)], inflammation status [assessed by C-reactive protein (CRP)] and oxidative stress [assessed by reactive oxygen species (ROS) and lag-time and slope]. It is speculated that oxidative stress increases RDW and, consequently, increases metabolic-related disorders, as assessed by the lipid profile [total cholesterol, high density lipoprotein (HDL) cholesterol, low density lipoprotein (LDL) cholesterol, and triglycerides], insulin, homeostatic model assessment (HOMA), and blood pressure in obese subjects (Fig. 2). These markers of metabolism, inflammation and oxidative stress were used due to previous investigations which reported elevated RDW being associated with high-sensitive CRP [12], altered glycemic patterns [13], hypertension [14], obesity [15] and unfavourable lipid profile, especially in women [16]. Finally, the core study hypothesis defines path effects of RDW and MCV on dysmetabolic biomarkers, by verifying their mediation roles in a pro-antioxidant balance (ROS-related) framework.

## Subjects and methods

### Subjects

The study was approved by the ethics committee of the Department of Internal Medicine and Medical Therapy at the University of Pavia, Italy (Reg. no 0905/15122017). All subjects gave their informed written consent to participate in the study, which was carried out in accordance with the Helsinki Declaration.

The subjects, both male and female, ranged in age between 18 and 50 years. Female subjects had to be premenopausal, not pregnant with normal menstrual cycles and having a BMI between 25 and 35 kg/m^2^. All subjects were asked to submit their complete medical history, and undergo a physical examination with anthropometric assessment and routine laboratory testing at the Dietetic and Metabolic Unit of the "Santa Margherita’ Institute, University of Pavia, Italy. Eligibility included subjects not showing significant alterations in lipid and carbohydrate metabolism (glucose < 6.11 mmol/L, total cholesterol < 6.20 mmol/L, triglycerides < 2.28 mmol/L) or being affected by any acute or disabling conditions or pathologic conditions (e.g., endocrinological, neoplastic and autoimmune diseases). Moreover, subjects with a history or signs and symptoms of heart disease were excluded. Subjects with mild hypertension (systolic pressure 140–150 mm/Hg, diastolic pressure 80–95 mm/Hg) and sedentary subjects, who did not drink more than 6 glasses of wine per week and no hard liquor were allowed in the study. Smoking habits were also recorded in a dichotomous way (yes or no) and previous smokers were considered as smokers.

### Observed variables

#### Nutritional status

Nutritional status was assessed using anthropometric measurements. Body weight was measured to the nearest 0.1 kg with a precision scale in light indoor clothing and without shoes, while WC was recorded to the nearest centimeter using standardized technique [17]. BMI was calculated (kg/m^2^).

#### Biochemical analyses

##### Lipid and glycemic pattern

Fasting venous blood samples were drawn from each subject in a sitting position between 08.00 and 10.00 AM. Blood collection and handling was performed under strictly standardized conditions and in line with manufacturer recommendations. Blood samples for clinical chemistry analysis were collected into evacuated tubes without anticoagulant, left for one hour at room temperature, and then centrifuged for 15 min at 1500 x g. The serum was then transferred into plastic tubes, rapidly frozen and stored at − 80 °C until analysis within one month. Whole blood with EDTA as anticoagulant was used for hematological indices. Total cholesterol, triglycerides and HDL cholesterol were measured by enzymatic-colorimetric methods. Serum LDL cholesterol was calculated according to the Friedewald formula for samples with triglyceride levels less than 400 mg/dL (< 4.5 mmol/L). Erythrocyte, white blood cell and platelet counts together with haemoglobin concentrations, mean cell volumes, and mean cell haemoglobin concentrations were measured using a Coulter automated cell counter MAX-M (Beckman Coulter, Inc., Fullerton, USA). This instrument takes advantage of the volume, conductivity and scatter (VCS) technology. Serum insulin levels were measured on a Roche Elecsys 2010 analyzer (Roche Diagnostics, Basel, Switzerland) using dedicated commercial electrochemiluminescent immunoassays. To determine insulin resistance, subjects were instructed to fast for 12 h before blood was taken. Furthermore, the subjects refrained from any form of exercise for 48 h before the study. Female subjects were tested during the early follicular phase of their menstrual cycles (days 3–10). Insulin resistance was calculated using the HOMA formula [18]: HOMA-IR = [(fasting insulin, μU/mL) x (plasma glucose, mmol/L)] divided by 22.5 [17].

#### Oxidative stress assessment

Whole blood with heparin as an anticoagulant was used to obtain plasma. The blood was immediately centrifuged (1000 x g for 15 min) and plasma aliquots were immediately frozen in liquid nitrogen and stored at − 80 °C until further assays. Red blood cells (RBCs), after buffy coat removal, were washed twice with phosphate buffered saline and aliquots were immediately frozen in liquid nitrogen and stored at − 80 °C until further assays.

The susceptibility of plasma to peroxidation was measured by determining the kinetics of copper-stimulated plasma peroxidation using a fluorescent method [19]. Briefly, this method is based on the evaluation of the peroxidation kinetics monitored following the formation of fluorescent adducts originating from the reaction of aldehydes (derived from lipid peroxidation promoted by Cu^++^ bound to apolipoproteins) with amino groups of plasma proteins and/or phospholipids. The development of fluorescence emission was monitored at 430 nm, setting the excitation at 360 nm, every 30 min for 8 h. The kinetic profile of these curves allows the evaluation of two indices of lipoprotein susceptibility to peroxidation: the lag-time of the initial latency phase expressed in minutes and the maximal rate of oxidation in the propagation phase calculated from the slope of the curve in this phase.

### Statistical analysis

The first aim of the research was to assess and quantify a causal path analysis model [20, 21] that describes the expected causal biomedical relationships between, adiposity indicators, such as BMI and WC, inflammation markers (i.e., CRP), ROS-related indicators (i.e., ROS, slope and lag-time), and blood indicators, such as RDW and MCV, and dysmetabolic markers as outcomes. The causal path diagram of the conceptual biomedical model is shown in Fig. 2, which is hierarchically structured by four sections: i) BMI and WC as antecedent nodes; ii) the inflammation markers, that are potentially first-level mediators; iii) the red blood markers, RDW and MCV that are potentially second-level mediators and finally iv) the dysmetabolic markers as outcomes.

Path analysis is a special type of structural equations modelling (SEM), a multivariate approach based on the use of a system of simultaneous equations to describe a priori path relationships that generate the data (SEMs belong to the confirmatory models class), where a given variable can appear explanatory in one or several equations, and as well as the outcome in other equations [20].

According to the conceptual model, the causal mechanism of a path analysis model defines 3 types of effects: direct, indirect, and correlation effect. The direct effect of an explanatory (exogenous) variable on a response (endogenous) variable is the net effect of a predictor, compared to the other predictors in the built-in equations. The indirect effect is the effect mediated by the other variables belonging to the pathway; these two are interpreted as a multiple linear regression coefficient (e.g., variation of response variable for unit increase of explanatory variable keeping fixed the others in the built-in equations). Finally, the correlation effect is defined by the classic Pearson correlation.

In this framework, by two tail z-tests on effects (null hypothesis, H_0_: β = 0, a *P*-value< 0.05 was considered significant), verification of (i) the direct effects of RDW and MCV on dysmetabolic markers as outcomes, (ii) whether adiposity markers affect RDW and MCV directly and/or indirectly via inflammation status, and (iii) whether RDW, MCV and inflammation markers could assume the role of mediators of the relationships between adiposity and dysmetabolic outcomes was possible. The residuals of the endogenous variables could be correlated (i.e. correlation coefficients = ρ), and they were interpreted as pairwise residual correlations.

Robust standard errors (sandwich type) were used to manage non-normality joint distribution. The authors evaluated the model fitting procedure by goodness-of-fit indexes such as variability explained of each response variable through the determination coefficients (R^2^), comparative fit index (CFI) and Tucker-Lewis index (TLI), and by badness-of fit indices such as root mean square error of approximation (RMSEA) and standardized root mean square residual (SRMR). CFI, TLI > 0.90, and RMSEA, SRMR< 0.08 were retained for “adequate approximation” fitting of the model to data.

The sample size (n = 166) was sufficient to fit the models because it is based on the ratio of sample size (N) to number of parameters (t) (i.e., N:t rule expected to be ≥2:1) to be estimated in SEM framework [22].

Finally, we performed a model selection procedure using a stepwise strategy by removing and adding new direct effects to the initial model. This modification was useful to refine the conceptual representation of the whole biomedical pathway. The criteria used for the model selection are based on the combination of two elements: (i) improvement of the previously mentioned indexes and (ii) z-tests of the maximum likelihood estimates (MLE) to detect the *P*-values < 0.10. However, from a statistical perspective, the authors acknowledge that once the conceptual model is revised the confirmatory modelling becomes exploratory modelling. Indeed, the data are random and the modifications based on the current sample may not be the modifications from another sample sampled under the same conditions. Hence, a further estimation procedure was tested, based on the semi-confirmatory approach via penalized likelihood [3, 6] by using the lasso penalization and the Akaike Information Criterion (AIC).

It is worth to point out that no extensive model searching has been done and the authors did not follow an exploratory approach. A stepwise regression model selection method was used to provide a potential and more consistent backbone model (supported by medical literature) that could be useful in future, larger studies and scenarios. In the end, the decision was to conduct a theory-driven study as much as possible relevant and concordant with the medical literature.

The path analysis parameters (effects) were computed by the maximum likelihood estimation (MLE) method. The *P*-values of the estimates were evaluated by z-tests (i.e., estimate over standard error), and were considered statistically significant if *P* < 0.05 in two sided tests. Standardized effects (β^*^) were also computed to compare the effect magnitudes (i.e., after adjustment for unit of measure of the variables). Statistical analysis was performed on R 3.6.3 using lavaan [23] and lslx [14] packages.

## Results

### Subject characteristics

**Fig. 1:**
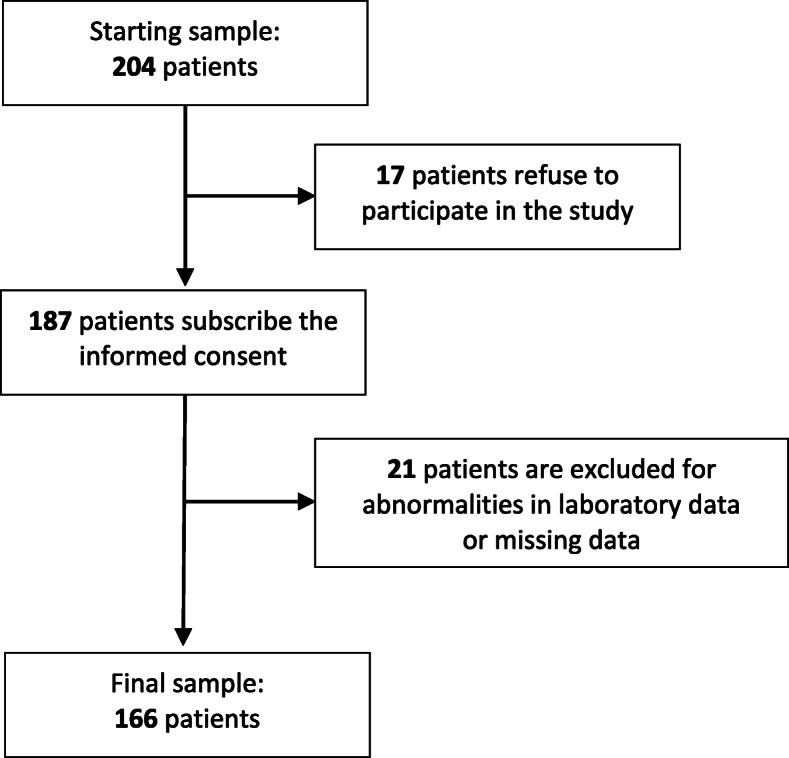
Flow diagram of the subjects studied

A total of 166 subjects (137 women and 29 men, including 38 ex- and current smokers), with a mean age of 39.38 ± 10.34 years admitted to the Dietetic and Metabolic Unit of the “Santa Margherita’ Institute, University of Pavia, Italy between January 2013 to the end of May 2015 were assessed. Figure 1 displays the subjects’ selection. General characteristics of the study population within gender are given in Table 1.

**Table 1:** Baseline descriptive statistics of the sample

Variable	Women (137)	Men (29)	Total (166)
	mean ± sd	mean ± sd	mean ± sd
age (years)	38.92 ± 10.48	41.55 ± 9.53	39.38 ± 10.34
**Adiposity markers**			
Body weight (kg)*	75.80 ± 10.11	94.90 ± 13.09	79.13 ± 12.89
Body Mass Index (kg/m^2^)*	29.70 ± 3.11	31.65 ± 3.67	30.04 ± 3.29
Waist circumference (cm)*	98.88 ± 8.96	109.31 ± 9.75	100.70 ± 9.91
**Inflammation markers**			
CRP (mg/dl) *	0.43 ± 0.52	0.26 ± 0.21	0.40 ± 0.48
Reactive Oxygen Species (mg/dl)*	31.71 ± 5.92	29.38 ± 3.57	31.30 ± 5.64
Slope (fluorescence units/min)	1.26 ± 0.28	1.23 ± 0.20	1.26 ± 0.26
Lag-time (min)	137.52 ± 18.86	141.90 ± 13.17	138.29 ± 18.04
**Red Blood markers**			
Red blood cell distribution width (%)*	14.48 ± 1.96	15.32 ± 1.32	14.63 ± 1.89
Mean Corpuscular Volume (femtoliters)	89.11 ± 3.84	89.39 ± 2.55	89.15 ± 3.65
**Dysmetabolic markers (outcomes)**			
Systolic Blood Pressure (mmHg)	132.89 ± 11.00	135.69 ± 7.27	133.38 ± 10.48
Diastolic Blood Pressure (mmHg)	81.52 ± 4.38	83.45 ± 5.01	81.86 ± 4.55
Blood glucose level (mmol/l)	5.01 ± 0.90	5.06 ± 0.59	5.02 ± 0.85
Insulin (mUI/L)	8.24 ± 3.69	9.07 ± 5.01	8.38 ± 3.95
HOMA (units)	1.81 ± 0.84	2.04 ± 1.16	1.85 ± 0.90
Triglycerides (mmol/l)	1.04 ± 0.53	1.27 ± 0.77	1.08 ± 0.58
Total Cholesterol (mmol/l)	5.27 ± 1.01	5.46 ± 1.14	5.31 ± 1.03
HDL Cholesterol (mmol/l)*	1.56 ± 0.36	1.36 ± 0.35	1.52 ± 0.37
LDL cholesterol (mmol/l)	3.11 ± 0.89	3.52 ± 1.04	3.19 ± 0.92
Total Cholesterol/HDL Cholesterol (units)*	3.45 ± 1.02	4.27 ± 1.44	3.60 ± 1.14

Data are expressed as mean ± standard deviation

*Statistically significant differences (T test or Mann-Whitney test, *P* < 0.05) between women and men

*CRP* C-Reactive Protein, *ROS* Reactive Oxygen Species, *HOMA* Homeostasis Model Assessment, *HDL* high-density lipoprotein cholesterol, *LDL* low-density lipoprotein cholesterol

### Path analysis model

Table 2 shows the estimates of the fit on the conceptual model. In order to achieve a more conservative and interpretable model, a first model selection procedure was performed using a backward technique where the unnecessary outcomes, whose regression coefficients returned *P*-value> 0.1, were removed. In this way, diastolic and systolic blood pressure, triglycerides, cholesterol, LDL cholesterol, and cholesterol/HDL cholesterol ratio were deleted from the model.

**Table 2:** Effect estimates of the path analysis of the conceptual model

	Estimate (β)	Standardized Estimate (β)^a^	P (|Z| > z)	95% CI
**Direct effect**				
BMI → CRP	**0.050**	**0.342**	**0.008**	**0.013; 0.087**
BMI → ROS	**0.415**	**0.242**	**0.047**	**0.006; 0.825**
BMI → Slope	− 0.012	− 0.153	0.192	− 0.031; 0.006
BMI → Lag-time	− 0.108	− 0.020	0.873	−1.432; 1.216
BMI → RDW	**−0.132**	**− 0.229**	**0.022**	**− 0.244; − 0.019**
BMI → MCV	0.078	0.070	0.574	−0.194; 0.350
WC → CRP	−0.002	−0.044	0.682	−0.012; 0.008
WC → ROS	−0.078	− 0.136	0.283	−0.220; 0.064
WC → Slope	0.000	0.003	0.975	−0.005; 0.006
WC → Lag-time	−0.292	− 0.160	0.143	− 0.684; 0.099
WC → RDW	**0.122**	**0.640**	**< 0.001**	**0.089; 0.155**
WC → MCV	−0.061	− 0.165	0.148	− 0.143; 0.022
CRP → RDW	*0.302*	*0.077*	*0.085*	*−0.042; 0.646*
CRP → MCV	**−1.446**	**−0.190**	**< 0.001**	**−2.170; − 0.721**
ROS → Slope	**0.012**	**0.263**	**0.006**	**0.004; 0.021**
ROS → Lag-time	**−1.276**	**−0.399**	**< 0.001**	**−1.867; − 0.685**
ROS → RDW	− 0.017	− 0.052	0.548	−0.074; 0.039
ROS → MCV	−0.003	− 0.005	0.943	− 0.097; 0.091
Slope→RDW	0.090	0.013	0.868	−0.971; 1.150
Slope→MCV	1.264	0.091	0.256	−0.917; 3.444
Lag-time→RDW	−0.001	−0.009	0.920	−0.019; 0.017
Lag-time→MCV	0.017	0.086	0.292	−0.015; 0.050
RDW → HOMA	**0.081**	**0.168**	**0.016**	**0.015; 0.146**
RDW → SBP	−0.206	−0.037	0.656	−1.115; 0.702
RDW → DBP	0.080	0.033	0.660	−0.277; 0.438
RDW → TGL	−0.007	−0.021	0.762	−0.049; 0.036
RDW → CHL	−0.009	−0.016	0.831	−0.089; 0.072
RDW → LDL	0.035	0.071	0.331	−0.036; 0.106
RDW → HDL	**−0.037**	**−0.192**	**0.007**	**−0.065; − 0.010**
RDW → CHL/HDL	*0.071*	*0.117*	*0.096*	*−0.012; 0.154*
RDW → BGL	*0.044*	*0.096*	*0.070*	*−0.004; 0.091*
RDW → Insulin	**0.320**	**0.153**	**0.030**	**0.030; 0.609**
MCV → HOMA	−0.028	−0.112	0.149	−0.066; 0.010
MCV → SBP	−0.081	−0.028	0.748	−0.576; 0.414
MCV → DBP	−0.071	−0.057	0.419	−0.244; 0.101
MCV → TGL	−0.013	−0.081	0.270	−0.036; 0.010
MCV → CHL	0.037	0.132	0.103	−0.008; 0.082
MCV → LDL	0.028	0.109	0.122	−0.007; 0.062
MCV → HDL	**0.014**	**0.136**	**0.048**	**0.000; 0.027**
MCV → CHL/HDL	−0.004	−0.014	0.834	−0.044; 0.035
MCV → BGL	0.007	0.029	0.514	−0.014; 0.027
MCV → Insulin	*−0.160*	*−0.148*	*0.050*	*−0.320; 0.000*

In **bold:*P*** **< 0.05;** in *italic: 0.05 ≤ P < 0.10*

Goodness of fit indexes: chi-square = 168.107 (P < 0.001), baseline chi-square = 1906.191 (P < 0.001), SRMR = 0.091, RMSEA =0.102 (90%CI = 0.084; 0.121), CFI = 0.940, TLI = 0.847

→ = effect, BMI = Body Mass Index, WC = Waist Circumference, CRP = C-Reactive Protein, ROS = Reactive Oxygen Species, RDW = Red Cell Distribution Width, MCV = Mean Corpuscular Volume, HOMA = Homeostasis Model Assessment, SBP = Systolic Blood Pressure, DBP = Diastolic Blood Pressure, TGL = Triglycerides, CHL = total cholesterol, LDL = low-density lipoprotein cholesterol, HDL = high-density lipoprotein cholesterol, BGL = Blood Glucose Level

^a^Since the variables are measured on various scales, standardized estimates (β*) rather than raw effects (β) are shown using the standard deviations as measurement units for the variables. Therefore, these are standardized partial regression coefficients are effects of explanatory variable on response variable, controlling for the other variables of the model

#### Goodness of fit indexes

The variability explained for each endogenous variable by its exogenous variables returned through the determination coefficients (R^2^) were in decreasing order: 25.2% for RDW, 21.3% for lag-time, 10.8% for CRP, 5.9% for slope, 5.2% for HDL cholesterol, 4.2% for MCV, 3.2% for HOMA, 2.9% for insulin, 1.8% for ROS and 0.9% for blood glucose levels. After model selection, the goodness-of-fit statistics as chi-square statistics = 88.839 (*P* < 0.001), baseline chi-square = 932.538 (*P* < 0.001), SRMR = 0.104, RMSEA = 0.084 (90%CI = 0.061; 0.107), CFI = 0.944, and TLI = 0.906 gave evidence of a moderate model appropriateness. All goodness of fit statistics was performed by the Satorra-Bentler correction.

#### Direct effects

The main output for the selected path analysis model is shown in Table 3. Notably, WC was linked to RDW as a red blood marker whereby, when WC increased, there was a corresponding increase in RDW (β = 0.123, *P* < 0.001). Moreover, RDW affected HDL cholesterol make it decrease (β = − 0.038, *P* = 0.006), and HOMA (β = 0.086, *P* = 0.010) and insulin (β = 0.343, *P* = 0.020) make them increase. Then, adiposity markers acted on inflammatory status. The results revealed that BMI was strongly linked to CRP (β = 0.048, *P* < 0.001), whereby an increase in BMI unit resulted in an increase in CRP (0.048 mg/dL), and ROS (β = 0.233, *P* = 0.049). In the same regards, WC significantly acted on lag-time (β = − 0.365, *P* < 0.001) with a 10-cm increase in WC resulting in a decreased lag-time of 3.2 min, which in turn led to the decision to communicate the value per 10 cm in accordance to size when interpreting the effect of WC.

**Table 3:** Effect estimates of the selected path analysis model (via stepwise regression)

	Estimate (β)	Standardized Estimate (β)*	P (|Z| > z)	95% CI
**Direct effect retained by model selection**				
BMI → CRP	**0.048**	**0.328**	**0.001**	**0.019; 0.078**
BMI → ROS	**0.233**	**0.136**	**0.049**	**0.001; 0.465**
BMI → RDW	**−0.138**	**− 0.241**	**0.012**	**− 0.247; − 0.030**
WC → Lag-time (^a^)	**− 0.365**	**− 0.198**	**< 0.001**	**− 0.549; − 0.180**
WC → RDW	**0.123**	**0.646**	**< 0.001**	**0.091; 0.156**
CRP → RDW	0.292	0.075	0.101	−0.057; 0.642
CRP → MCV	**−1.546**	**−0.205**	**< 0.001**	**− 2.317; − 0.775**
ROS → Slope	**0.011**	**0.243**	**0.010**	**0.003; 0.020**
ROS → Lag-time	**−1.278**	**−0.396**	**< 0.001**	**− 1.860; − 0.696**
RDW → HOMA	**0.086**	**0.179**	**0.010**	**0.021; 0.151**
RDW → HDL	**−0.038**	**− 0.196**	**0.006**	**−0.065; − 0.011**
RDW → BGL	*0.042*	*0.094*	*0.074*	*−0.004; 0.089*
RDW → Insulin	**0.343**	**0.165**	**0.020**	**0.055; 0.631**
MCV → HDL	0.011	0.113	0.105	−0.002; 0.025
MCV → Insulin	**−0.036**	**−0.033**	**0.003**	**−0.060; − 0.012**
**(main) indirect effect**				
BMI → CRP → MCV	**− 0.075**	**−0.067**	**0.012**	**−0.133; − 0.016**
BMI → ROS → Lag-time	*−0.298*	*− 0.054*	*0.070*	*− 0.620; 0.024*
BMI → RDW → HOMA	*−0.012*	*− 0.043*	*0.056*	*− 0.024; 0.000*
BMI → RDW → HDL	*0.005*	*0.047*	*0.050*	*0.000; 0.010*
BMI → RDW → Insulin	*−0.047*	*−0.040*	*0.069*	*−0.098; 0.004*
WC → RDW → HOMA	**0.011**	**0.116**	**0.013**	**0.002; 0.019**
WC → RDW → HDL	**−0.005**	**−0.126**	**0.010**	**−0.008; − 0.001**
WC → RDW → BGL	*0.005*	*0.061*	*0.084*	*−0.001; 0.011*
WC → RDW → Insulin	**0.042**	**0.107**	**0.024**	**0.005; 0.079**
CRP → MCV → Insulin	**0.055**	**0.007**	**0.015**	**0.011; 0.100**

In **bold: P < 0.05;** in *italic: 0.05 ≤ P < 0.10*

Goodness of fit indexes: chi-square = 88.839 (P < 0.001), baseline chi-square = 932.538 (P < 0.001), SRMR = 0.104, RMSEA = 0.084 (90%CI = 0.061; 0.107), CFI = 0.944, TLI = 0.906

(^a^) discovered by the modification indexes

→ = effect, BMI = Body Mass Index, CRP = C-Reactive Protein, ROS = Reactive Oxygen Species, RDW = Red Cell Distribution Width, MCV = Mean Corpuscular Volume, WC = Waist Circumference, HOMA = Homeostasis Model Assessment, HDL = high-density lipoprotein cholesterol, BGL = Blood Glucose Level

*Since the variables are measured on various scales, standardized estimates (β*) rather than raw effects (β) are shown using the standard deviations as measurement units for the variables. Therefore, these are standardized partial regression coefficients are effects of explanatory variable on response variable, controlling for the other variables of the model

Regarding the inflammation pathways, Table 3 also shows a significant effect of CRP on MCV (β = − 1.546, *P* < 0.001) in which MCV decreased by 1.546 femtoliters for each unit of CRP increase. Moreover, ROS-related process showed significant effects of ROS on both lag-time (β = − 1.278, *P* < 0.001) and slope (β = 0.011, *P* = 0.010). For every ten units of ROS increase, lag-time decreased by 12.78 min, while slope increased by 0.11 fluorescence units/min. Notably, the effects linking the ROS-related markers to RDW and MCV were all non-significant (*P* > 0.05). Finally, MCV affected insulin (β = − 0.036, *P* = 0.003).

In order to compare the main effects, the standardized versions were evaluated as shown in Table 3. Accounting for this, regarding the significant ones, the effect of WC on RDW (in absolute value) was the strongest (β* = 0.646) of the selected path analysis model. In addition, the effect of ROS on lag-time was very marked (β* = − 0.396) as well as the BMI effect on CRP (β* = 0.328).

#### Indirect effects

Table 3 displays the significant indirect effects, computed as product of the direct effects involved. These effects set shown as RDW might be considered a mediator of the relationships of WC on some dysmetabolic outcomes. In particular, WC affected RDW (β = 0.123) with the latter being linked negatively to the three dysmetabolic outcomes, i.e., decreased HDL cholesterol (β = − 0.005 = 0.123 × − 0.038, *P* = 0.010), increased HOMA (β = 0.011 = 0.123 × 0.086, *P* = 0.013) and insulinemia (β = 0.042 = 0.123 × 0.343, *P* = 0.024).

Finally, BMI-induced CRP negatively affected MCV in a significant manner (β = − 0.075 = 0.048 × − 1.546, *P* = 0.012), and CRP affected insulin by MCV in damaging way (β = 0.055 = 1.546 × − 0.036, *P* = 0.015).

#### Residual correlations

The residual correlation (ρ) estimates of the endogenous variables are shown in the upper triangular matrix section of Table 4. Accounting for outcome section, insulin and HOMA residuals were strongly correlated between them (ρ = 0.981, *P* < 0.001). Regarding the other two model sections, i.e., inflammation and red blood markers, it should be noted that the slope-lag-time and the slope-CRP residual correlations were slightly significant, ρ = − 0.233 (*P* = 0.013) and ρ = 0.160 (*P* = 0.033), respectively.

**Table 4:** Coefficients of determination (R2) and residual correlations (ρ) by section of the selected path analysis model (via stepwise regression)

		Inflammation markers (first level mediators)				Red Blood markers (second level mediators)		Outcomes			
		CRP	ROS	Slope	Lag-time	RDW	MCV	Insulin	HOMA	HDL	BGL
**Inflammationmarkers** **(first level mediators)**	**CRP**	**0.108** **(P = 0.027)**	0.097 (P = 0.294)	**0.160** **(P = 0.033)**	−0.020 (P = 0.708)	–	–	–	–	–	–
	**ROS**		**0.018** **(P < 0.001)**	–	–	–	–	–	–	–	–
	**Slope**			**0.059** **(P < 0.001)**	**−0.233** **(P = 0.013)**	–	–	–	–	–	–
	**Lag-time**				**0.213** **(P < 0.001)**	–	–	–	–	–	–
**Red Blood-markers (second level mediators)**	**RDW**					**0.252** **(P < 0.001)**	0.000 (P = 0.998)	–	–	–	–
	**MCV**						**0.042** **(P < 0.001)**	–	–	–	–
**Outcomes**	**Insulin**							**0.029** **(P < 0.001)**	**0.981** **(P < 0.001)**	**−0.171** **(P = 0.040)**	0.021 (P = 0.629)
	**HOMA**								**0.032** **(P < 0.001)**	*−0.157* *(P = 0.055)*	**0.141** **(P = 0.003)**
	**HDL**									**0.052** **(P < 0.001)**	0.104 (P = 0.293)
	**BGL**										0.009 (P = 0.107)

R^2^’s are on the diagonal, residual correlations (ρ) in the upper triangular matrix (**in bold: P < 0.05**)

*CRP* C-Reactive Protein, *ROS* Reactive Oxygen Species, *RDW* Red Cell Distribution Width, *MCV* Mean Corpuscular Volume, *HOMA* Homeostasis Model Assessment, *HDL* high-density lipoprotein cholesterol, *BGL* Blood Glucose Level

#### Semi-confirmatory approach

**Fig. 2:**
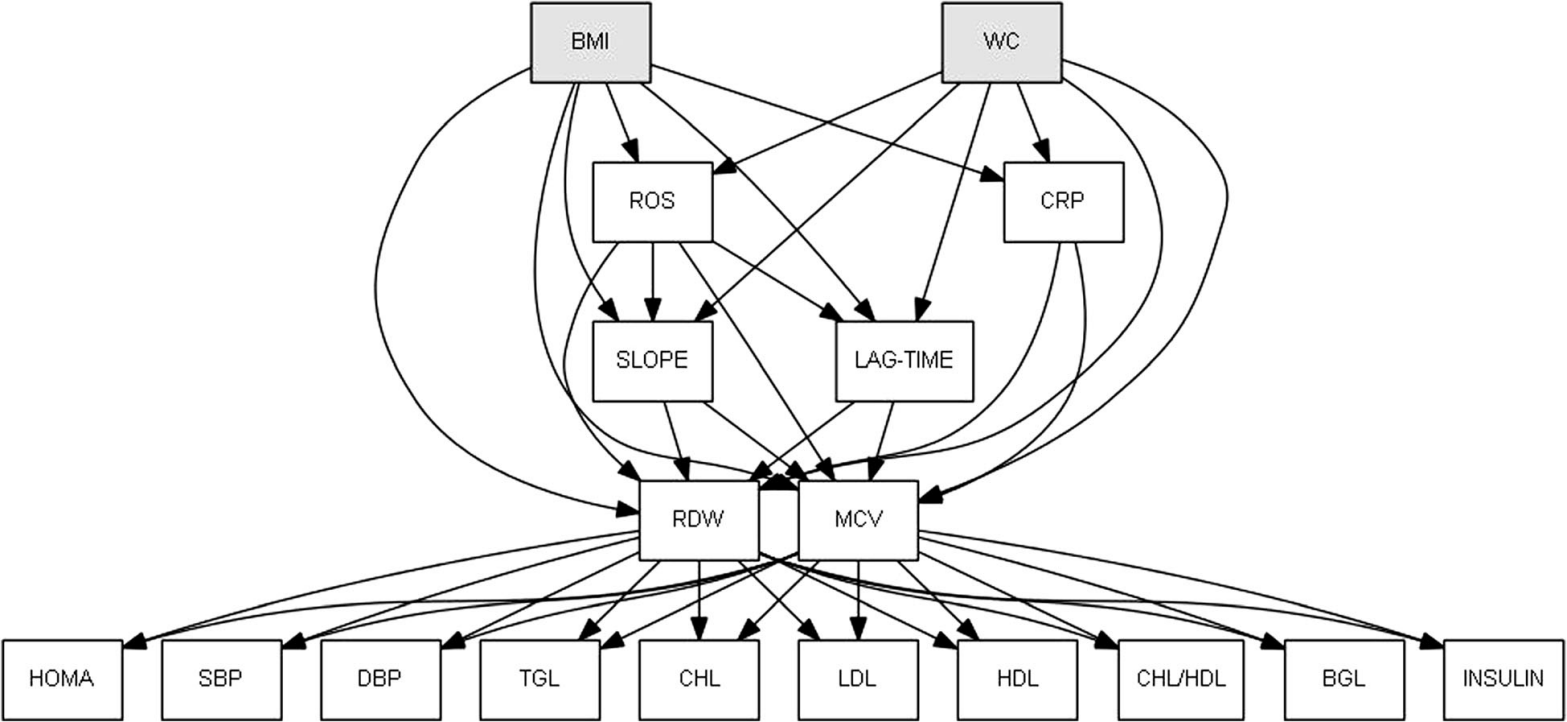
Graph of the conceptual path analysis model. Note. → = effect, BMI = Body Mass Index, WC = Waist Circumference, CRP = C-Reactive Protein, ROS = Reactive Oxygen Species, RDW = Red Cell Distribution Width, MCV = Mean Corpuscular Volume, SBP = Systolic Blood Pressure, DBP = Diastolic Blood Pressure, BGL = Blood Glucose Level, HOMA = Homeostasis Model Assessment, TGL = Triglycerides, CHL = cholesterol, HDL= high-density lipoprotein cholesterol, LDL = low-density lipoprotein cholesterol. The grey boxes represent the exogenous variables, the white boxes the endogenous ones. All the variables are continuous

Following this approach, the model selection might also suggest the inclusion of effects (β_PL_) that were not stated in the conceptual model (Fig. 2), that may explain more variability, in addition to improving adequacy of the model to data. In this way, the direct effects of the adiposity on the dysmetabolic outcomes were probed, accounting for the small sample size of the study. In this case, overall, the goodness of fit indexes were not satisfactory by giving evidence of a poor model appropriateness (data not shown). Nevertheless, it is worth reporting the significant results on the new direct effects inserted by this approach: BMI had a significant effect on insulin (β_PL_ = 0.281, *P* = 0.023, 95%CI = 0.005; 0.558) and HOMA (β_PL_ = 0.086, *P* = 0.004, 95%CI = 0.023; 0.150), while WC had an effect on LDL cholesterol (β_PL_ = 0.022, *P* = 0.049, 95%CI = 0.000; 0.047), triglycerides (β_PL_ = 0.025, *P* = 0.001, 95%CI = 0.009; 0.041) and the total cholesterol – HDL cholesterol ratio (β_PL_ = 0.04, *P* < 0.001, 95%CI = 0.022; 0.058).

## Discussion

This study was conducted in a group of obese subjects using a path analysis model that characterizes and quantifies pathways of adiposity and inflammation on dysmetabolic biomarkers through red blood indicators such as RDW and MCV.

Recently, RDW has been suggested to have an important role in the final common pathway of multiple pathologic processes, including inflammation and therefore reflecting different forms of anaemia [4].

The authors therefore state that elevated measures of RDW represent the underlying inflammatory state in overweight subjects, which is consistent with the current understanding of the crosstalk between inflammation and the hematological system. It is well established that inflammatory cytokines interfere with the maturation of RBCs in the bone marrow through multiple mechanisms [24–27].

In addition, a moderate prediction was found for RDW (25.2%), lag-time (21.3%) and CRP (10.8%). On the other hand, the main strength of this study was to investigate the mechanism describing the causal relationships of key metabolic biomedical markers, which have been separately studied in the literature. They were included in the hierarchical multivariate model that tested the effects on dysmetabolic outcomes. These factors combined with rigorous inclusion and exclusion criteria provided by the model selection used improved the quality of the results obtained with respect to previous literature.

Furthermore, this study demonstrated the potentially adverse effects of BMI on inflammation markers, as witnessed by the elevated serum levels of CRP and ROS. Additionally, BMI appears to have an indirect effect on MCV, whereby the BMI-induced increases in CRP may have generated a negative trickle-down effect leading to a reduction in MCV. Emerging data from this study supplements already existing data from studies that previously demonstrated a strong association between RDW and CRP [12]. Moreover, a number of studies have reported a correlation in decreased MCV with inflammation markers in patients with inflammatory bowel disease [28, 29].

The findings of this study showed a direct association between WC and RDW, which is in line with previous studies that reported significantly higher mean platelet volume and RDW in patients with adiposity related MetS compared to those without MetS or positively correlated with high-sensitive CRP, HOMA insulin resistance and BMI [30, 31].

As a result, the effect of RDW may therefore be related to the reduction of HDL cholesterol and the increase in HOMA and insulin values. In particular, the possible consequences of the indirect effect involving adiposity, RDW and HDL cholesterol may be related to cardiovascular diseases. Indeed, an association between RDW and lipid profile has been observed in previous studies showing an inverse relationship between RDW and all coronary artery diseases and plasma lipids [16, 32]. Finally, WC values showed an association with the inflammation marker of lag-time.

It is worth mentioning that the authors of this study have considered the effects of ROS on lag-time. The findings showed a strong negative effect between the increase in ROS and the decrease in lag-time. In addition, ROS had a strong positive effect on the slope with increasing ROS corresponding to increased slope. Nevertheless, it must be pointed out that ROS-related markers had no effect on RDW and MCV. Therefore, ROS had no indirect side effects on the metabolic markers.

### Study limitations

The results of this study should be evaluated considering that the main limitation of this study is the relatively small size of the sample studied. Therefore, further studies, employing larger number of subjects of both sexes, may provide more detailed information on the potential direct links between inflammatory status (e.g., ROS-related markers) and dysmetabolic outcomes. In addition, adjustment in covariates or confounding might be involved to refine the parameter estimates.

## Conclusion

The present study offers an innovative model that is directly involved in the assessment about the effects of red blood indicators, specifically RDW and MCV, as suitable markers for cardiovascular disease (HDL cholesterol), inflammatory bowels and insulin resistance. In a group of obese subjects, this statistical model was able to characterizes and quantifies pathways of adiposity and inflammation on dysmetabolic biomarkers through RDW and MCV. In particular, RDW may be considered a mediator in the relationships between WC and the dysmetabolic outcomes, whereas CRP seems to modulate the linkage between BMI and MCV. Further researches are necessary to better investigated the role of blood indicators on dysmetabolic outcomes.

## Data Availability

Not applicable.

## Abbreviations

RDW: Red blood cell distribution width
CHF: Including chronic heart failure
MetS: Metabolic syndrome
ICARIA: Ibermutuamur CArdiovascular RIsk Assessment study
ROS: Reactive oxygen species
BMI: Body mass index
WC: waist circumference
CRP: C reactive protein
WC: Waist circumference
HOMA: Homeostasis Model Assessment
RBCs: Red blood cells
SEM: Structural equations modelling
CFI: Comparative fit index
TLI: Tucker-Lewis index
RMSEA: Root mean square error of approximation
SRMR: Standardized root mean square residual
MLE: Maximum likelihood estimates

## References

[1] Patel KV, Semba RD, Ferrucci L, Newman AB, Fried LP, Wallace RB, Bandinelli S, Phillips CS, Yu B, Connelly S, Shlipak MG, Chaves PHM, Launer LJ, Ershler WB, Harris TB, Longo DL, Guralnik JM (2010). Red Cell distribution width and mortality in older adults: A meta-analysis. J Gerontol - Ser A Biol Sci Med Sci.

[2] Jung C, Fujita B, Lauten A, Kiehntopf M, Küthe F, Ferrari M, Figulla H-R (2011). Red blood cell distribution width as useful tool to predict long-term mortality in patients with chronic heart failure. Int J Cardiol.

[3] Sadaka F, O’Brien J, Prakash S (2012). Red cell distribution width and outcome in patients with septic shock. J Intensive Care Med.

[4] Allen LA, Felker GM, Mehra MR, Chiong JR, Dunlap SH, Ghali JK, Lenihan DJ, Oren RM, Wagoner LE, Schwartz TA, Adams KF (2010). Validation and potential mechanisms of red cell distribution width as a prognostic marker in heart failure. J Card Fail.

[5] Förhécz Z, Gombos T, Borgulya G, Pozsonyi Z, Prohászka Z, Jánoskuti L (2009). Red cell distribution width in heart failure: prediction of clinical events and relationship with markers of ineffective erythropoiesis, inflammation, renal function, and nutritional state. Am Heart J.

[6] Sanchez-Chaparro MA, Calvo-Bonacho E, Gonzalez-Quintela A, Cabrera M, Sainz JC, Fernandez-L C, Aguado LQ, Meseguer AF, Valdivielso P, Roman-Garcia J (2010). OBSERVATIONS Higher Red Blood Cell Distribution Width Is Associated With the Metabolic Syndrome.

[7] Kalupahana NS, Moustaid-Moussa N, Claycombe KJ (2012). Immunity as a link between obesity and insulin resistance. Mol Asp Med.

[8] Fujita B, Strodthoff D, Fritzenwanger M, Pfeil A, Ferrari M, Goebel B, Figulla HR, Gerdes N, Jung C (2013). Altered red blood cell distribution width in overweight adolescents and its association with markers of inflammation. Pediatr Obes.

[9] Vayá A, Alis R, Hernandez-Mijares A, Solá E, Cámara R, Rivera L, Romagnoli M, Laiz B (2014). Red blood cell distribution width is not related with inflammatory parameters in morbidly obese patients. Clin Biochem.

[10] Semba RD, Patel KV, Ferrucci L, Sun K, Roy CN, Guralnik JM, Fried LP (2010). Serum antioxidants and inflammation predict red cell distribution width in older women: the Women’s health and aging study I. Clin Nutr.

[11] Ghaffari S (2008). Oxidative stress in the regulation of normal and neoplastic hematopoiesis. Antioxid Redox Signal.

[12] Lippi G, Targher G, Montagnana M, Salvagno GL, Zoppini G, Guidi GC (2009). Relation between red blood cell distribution width and inflammatory biomarkers in a large cohort of unselected outpatients. Arch Pathol Lab Med.

[13] Veeranna V, Zalawadiya SK, Panaich SS, Ramesh K, Afonso L (2012). The association of red cell distribution width with glycated hemoglobin among healthy adults without diabetes mellitus. Cardiology..

[14] Tanindi A, Topal FE, Topal F, Celik B (2012). Red cell distribution width in patients with prehypertension and hypertension. Blood Press.

[15] Perlstein TS, Weuve J, Pfeffer MA, Beckman JA (2009). Red blood cell distribution width and mortality risk in a community-based prospective cohort. Arch Intern Med.

[16] Lippi G, Sanchis-Gomar F, Danese E, Montagnana M (2013). Association of red blood cell distribution width with plasma lipids in a general population of unselected outpatients. Kardiol Pol.

[17] Frisancho AR (1984). New standards of weight and body composition by frame size and height for assessment of nutritional status of adults and the elderly. Am J Clin Nutr.

[18] Haffner SM, Kennedy E, Gonzalez C, Stern MP, Miettinen H (1996). A prospective analysis of the HOMA model. The Mexico City diabetes study. Diabetes Care.

[19] Cazzola R, Russo-Volpe S, Miles EA, Rees D, Banerjee T, Roynette CE, Wells SJ, Goua M, Wahle KWJ, Calder PC, Cestaro B (2007). Age- and dose-dependent effects of an eicosapentaenoic acid-rich oil on cardiovascular risk factors in healthy male subjects. Atherosclerosis..

[20] Bollen K (2014). Structural equations with latent variables.

[21] Pearl J (2014). Interpretation and identification of causal mediation. Psychol Methods.

[22] Bagozzi RP, Yi Y (2012). Specification, evaluation, and interpretation of structural equation models. J Acad Mark Sci.

[23] Rosseel Y. lavaan: an R package for structural equation modeling and more Version 0.5–12 (BETA) [Internet].

[24] Chiari MM, Bagnoli R, De Luca P, Monti M, Rampoldi E, Cunietti E (1995). Influence of acute inflammation on Iron and nutritional status indexes in older inpatients. J Am Geriatr Soc.

[25] Jelkmann WEB, Fandrey J, Frede S, Pagel H (1994). Inhibition of erythropoietin production by cytokines: implications for the Anemia involved in inflammatory states. Ann N Y Acad Sci.

[26] Macdougall IC, Cooper A (2002). The inflammatory response and epoetin sensitivity. Nephrol Dial Transplant.

[27] Nicolas G, Chauvet C, Viatte L, Danan JL, Bigard X, Devaux I, Beaumont C, Kahn A, Vaulont S (2002). The gene encoding the iron regulatory peptide hepcidin is regulated by anemia, hypoxia, and inflammation. J Clin Invest.

[28] Cakal B, Akoz AG, Ustundag Y, Yalinkilic M, Ulker A, Ankarali H (2009). Red cell distribution width for assessment of activity of inflammatory bowel disease. Dig Dis Sci.

[29] Thomas CW, Lowry PW, Franklin CL, Weaver AL, Myhre GM, Mays DC, Tremaine WJ, Lipsky JJ, Sandborn WJ (2003). Erythrocyte mean corpuscular volume as a surrogate marker for 6-thioguanine nucleotide concentration monitoring in patients with inflammatory bowel disease treated with azathioprine or 6-mercaptopurine. Inflamm Bowel Dis.

[30] Farah R, Khamisy-Farah R (2015). Significance of MPV, RDW with the presence and severity of metabolic syndrome. Exp Clin Endocrinol Diabetes.

[31] Yilmaz Ö, Mehmet C, Kelekci S, Temur M (2015). Association between red blood cell distribution width and polycystic ovary syndrome. Endocr Res.

[32] Gürel O, Demircelik M, Bilgic M, Yilmaz H, Yilmaz O, Cakmak M, Eryonucu B (2015). Association between red blood cell distribution width and coronary artery calcification in patients undergoing 64-multidetector computed tomography.

